# A Method for Quantitative Determination of Biofilm Viability

**DOI:** 10.3390/jfb3020418

**Published:** 2012-06-01

**Authors:** Ken Welch, Yanling Cai, Maria Strømme

**Affiliations:** Division for Nanotechnology and Functional Materials, Department of Engineering Sciences, The Ångström Laboratory, Uppsala University, Box 534, 75121 Uppsala, Sweden; Email: yanling.cai@angstrom.uu.se

**Keywords:** specific growth rate, metabolic activity assay, biofilm, *Streptococcus mutans*, phenol red

## Abstract

In this study we present a scheme for quantitative determination of biofilm viability offering significant improvement over existing methods with metabolic assays. Existing metabolic assays for quantifying viable bacteria in biofilms usually utilize calibration curves derived from planktonic bacteria, which can introduce large errors due to significant differences in the metabolic and/or growth rates of biofilm bacteria in the assay media compared to their planktonic counterparts. In the presented method we derive the specific growth rate of *Streptococcus mutans* bacteria biofilm from a series of metabolic assays using the pH indicator phenol red, and show that this information could be used to more accurately quantify the relative number of viable bacteria in a biofilm. We found that the specific growth rate of *S. mutans* in biofilm mode of growth was 0.70 h^−1^, compared to 1.09 h^−1^ in planktonic growth. This method should be applicable to other bacteria types, as well as other metabolic assays, and, for example, to quantify the effect of antibacterial treatments or the performance of bactericidal implant surfaces.

## 1. Introduction

Bacterial biofilms are structures consisting of extracellular polymeric substances and single or multiple species of bacteria that form clusters and adhere to surfaces [1,2]. A major proportion of the biofilm, up to 97%, consists of bound water [3]. Compared to planktonic bacteria, bacteria in biofilms better survive harsh environmental conditions, and consequently biofilm bacteria show a 10–1,000 times higher resistance towards antibiotics than planktonic bacteria [4]. This has become a major healthcare concern as antibiotic-resistant bacterial strains like methicillin-resistant *Staphylococcus aureus* (MRSA) are appearing. Multiple factors contribute to this resistance, including physical and chemical diffusion barriers [5], reduced sensitivity towards antibiotics due to the slow growth rate of bacteria in biofilms [6,7,8], as well as the structural heterogeneity within biofilms and development of biofilm-specific biocides-resistant bacteria phenotypes [4,9]. Furthermore, biofilms are often associated with implants or other biomaterials as such biocompatible materials also provide an ideal substrate for biofilm formation. Understanding biofilms and the antibacterial methods for their removal or inhibition is thus critical in the development of functional biomaterials.

To be able to determine the microbial antibiotic susceptibility, or assess the effectiveness of other bactericidal treatments on bacteria in biofilm, it is essential to be able to determine the amount of viable bacteria in the biofilm, or at least the relative reduction in viable bacteria due to the treatment. There are several means for quantification of bacterial viability, but these are generally much better suited for evaluating planktonic cultures. Unfortunately, one cannot transfer results from tests performed on planktonic bacteria to biofilm bacteria since it is known that, for example, bacteria in biofilm have an inherent lack of susceptibility to antibiotics compared to planktonic cultures of the same bacteria. The classical means of determining bacterial viability is counting the number of colony forming units (cfu) after plating cultures. Using this method to assess biofilm viability can lead to significant errors, since there is a high degree of aggregation due to the presence of the biofilm matrix. Procedures such as sonication or the use of matrix degrading enzymes can be used to separate bacteria from the matrix or the surface to which they are attached, but have the disadvantage that the viability of the bacteria may be affected. Additionally, the bacteria cells must be re-suspended in order to perform the cfu counting. The Calgary Biofilm Device [10] is a popular method that utilizes such procedures in determining the microbial antibiotic susceptibility of bacteria in biofilm, but does not detect the number of bacteria in the biofilm. Staining techniques such as crystal violet or live/dead staining [11] provide valuable information, but also have inherent limitations. Crystal violet provides a good measure of biofilm mass; however, it does not give a measure of biofilm viability as it stains both the bacteria cells and the extracellular matrix. Live/dead staining is used to quantify planktonic bacteria in combination with, for example, flow cytometry [11], and is useful for imaging biofilms, but is not ideal for high-throughput quantification of biofilm viability as it must be used in conjunction with a laser scanning confocal microscope in a time consuming process where only a small section of the biofilm can be assessed at a time.

Metabolic assays are excellent candidates for quantification of bacterial viability in biofilm. These assays are indirect methods based on the detection of metabolic products produced by bacteria and have the advantage of being able to assess viability without sample manipulation since these assays generally do not require the removal of the biofilm from the adherent surface. A number of different indicators are used for the detection of bacterial metabolic activity. For example, the resazurin assay (also known as the Alamar Blue assay) is based on the reduction of resazurin, a blue dye that can be reduced by metabolically active cells to pink resorufin, which is fluorescent [12]. Thus, fluorescent measurements of a resazurin assay containing a biofilm can be used to quantify the viability of the biofilm [13,14]. Similarly, an assay based on the conversion of non-fluorescent fluorescein diacetate (FDA) into the highly fluorescent fluorescein has been used for the quantification of biofilm mass [15,16] and viability [17]. Another strategy is the use of a pH indicator to measure the change in pH of a viability assay due to the production of acids by bacterial metabolism [18,19]. Part of the reason for the existence of the variety of metabolic assays is the fact that often the assays are strain specific or work better with certain types of bacteria.

However, a significant limitation of these metabolic assays that has not been adequately addressed is the fact that bacteria in biofilm do not have the same metabolic activity as planktonic bacteria. In order to correlate the metabolic signal of the assay containing the biofilm in question, one must know how many viable bacteria in such a biofilm produce a given signal. Typically, assays performed on biofilm are calibrated against their planktonic cultures, with the assumption that the metabolic rates are similar. However, this introduces significant errors since it is known that the metabolic rates of bacteria can differ greatly between their planktonic and biofilm form [4,20,21]. For example, it has been shown that when using the resazurin metabolic assay, *Staphylococcus aureus* planktonic bacterial suspensions generate a larger signal than that from the same concentration of corresponding biofilm bacteria [22]. 

We present a scheme for quantitative determination of biofilm viability and show that this information could be used to determine, for example, the effect of antibacterial treatments or the performance of a bactericidal implant surface [23,24,25]. The specific growth rate of biofilm bacteria is determined by a series of metabolic assays, and this information is used to calibrate the results of biofilm bacteria viability testing. We apply the method for quantification of a *Streptococcus mutans* biofilm.

## 2. Theory

A metabolic assay is used to assess the number of viable bacteria in the assay media through the production of metabolites that are subsequently detected or measured. Normally the relationship between the starting bacterial population and the signal level (or time to achieve a certain signal level) in an assay is empirically determined by producing a standard curve from a series of bacteria cultures with different starting concentrations. This poses no difficulty with planktonic bacterial cultures since it is easy to determine the starting concentration; however, this is not the case with bacteria in biofilm form since it is practically impossible to know the starting number of viable bacteria in a biofilm. 

The amount of metabolite produced by a biofilm, or a planktonic culture, in a viability assay will depend on both the metabolic activity of the individual bacteria and the number of bacteria. If the assay indicator (e.g., color of the pH indicator or the amount of fluorescence) is measured shortly after the biofilm is placed in the assay, the measurement corresponds to the initial number of viable bacteria in the biofilm since the bacteria have not had time to multiply. However, signal levels may be too small to be detected reliably if the bacteria population is relatively small. The detection range of the method can be greatly extended by allowing the culture to multiply until the population of bacteria is large enough to produce a measureable signal. In this case, the growth rate of the bacteria plays an important part in the measured signal. An example of this type of viability assay is one incorporating phenol red, a pH indicator that changes the color of the assay media when the bacteria have produced enough acid metabolites.

An analytic estimation of the time for a bacterial culture to produce a certain signal level can be determined by the following analysis. In this analysis, we consider the assay to contain an excess of sustenance that allows the bacteria to exist in the exponential growth phase. In this case, we can represent the number of bacteria at time *t* by the Equation (1) 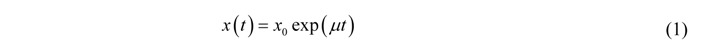 Where 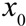 is the initial number of bacteria, 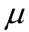 is the specific growth rate, and the time for the bacterial population to double (*i.e.*, the generation time) is given by 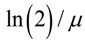.

If 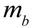 is the amount of metabolite produced per bacterium per unit of time, then 
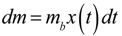
 is the amount of metabolite produced in the time interval *dt* at time *t*. In order to calculate the accumulated amount of metabolite at time *t* we integrate *dm*: 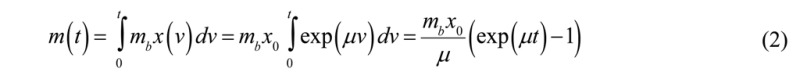 where *v* is the integration variable. If the 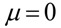 (*i.e.*, the bacterial population is constant), then Equation (2) simplifies to 
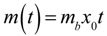
 as expected (this can be proved by taking the Maclaurin series of 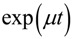 before setting 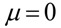 in Equation (2)).

If a certain amount of metabolite is required to produce a signal (e.g., a specific amount of metabolite is required to change the color of the phenol red assay to yellow) we can write the relationship between the time, 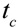, it takes a starting amount of bacteria, 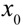, to produce the required amount of metabolite, 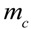: 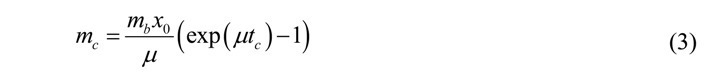 solving for 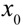 gives 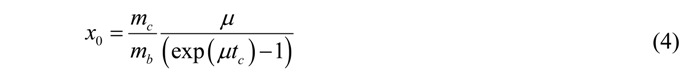 for 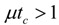, 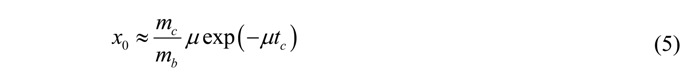


Equation (5) can also be expressed in terms of 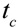: 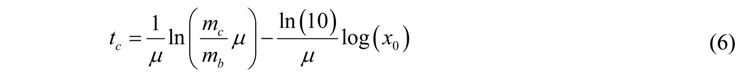


Thus, if we plot 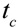 as a function of 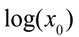 we find that the slope of the line corresponds to the inverse of the specific growth rate of the bacteria. This plot corresponds to the standard curves that are used to calibrate metabolic viability assays; different known starting concentrations of bacteria are placed in the assay and the times for the required amount of metabolite to be produced are recorded and plotted against the starting concentrations of bacteria. In fact, if we look at standard curves that have been produced in other studies with viability assays incorporating indicators such as phenol red or resazurin [14,19], we can observe the logarithmic relationship between time and initial bacterial concentration as indicated by Equation (6).

The fact that we show that the slope of the line in the standard curve is directly related to the specific growth rate suggests that these viability assays can be calibrated by other means that provide the specific growth rate of the bacteria in the assay media. With only knowledge of the bacterial growth rate, we cannot correlate the result of a viability assay to an exact number of viable bacteria; however, we can use it to quantify the relative number of viable bacteria between different samples. This would be of great use in determining the log reduction in antibiotic susceptibility testing or other types of antimicrobial treatments. 

For planktonic bacterial suspensions one could use turbidity (optical density, OD) measurements to determine the specific growth rate, since this is a standard method of determining the specific growth rate of bacteria cultures. One could argue, however, that this is unnecessary since a standard curve can be readily prepared with know bacterial concentrations when dealing with planktonic bacteria. However, this is not the case with biofilm since it is practically impossible to know the starting number of viable bacteria in a biofilm. Instead, one could perform a series of assays with, for example, a varying number of sample disks covered with biofilm. Plotting this relative number of biofilm bacteria versus the time required to produce the required signal would allow one to calculate the specific growth rate using Equation (6), and thus be able to quantitatively determine the log reduction in, for example, antibiotic susceptibility tests.

**Figure 1:**
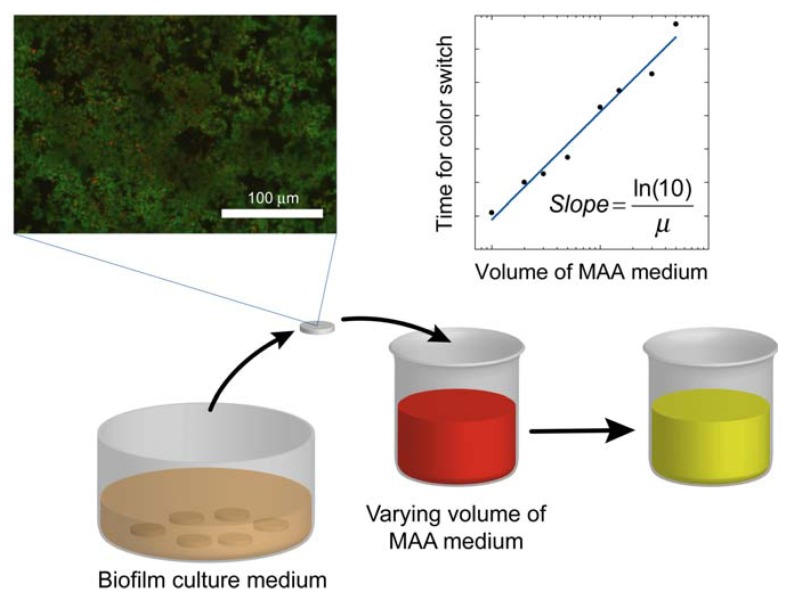
Schematic diagram of the method for determining the specific growth rate of biofilm bacteria. The specific growth rate *μ* is extracted from a plot of the time required for color switch of Metabolic Activity Assay (MAA) broth containing a set amount of biofilm bacteria as a function of MAA broth volume. Included is a laser scanning confocal microscopy image of a *S. mutans* biofilm using live/dead staining (L13152, Invitrogen, Eugene, UR, USA).

If the above technique using a varying number of sample surfaces covered with biofilm is not possible or practical, another means of determining the specific growth rate is required. Returning to Equation (3), we can see that for a fixed initial number of bacteria 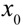 there is an exponential relationship between the amount of metabolite required to produce a signal and the corresponding time 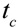 for values of 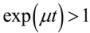. Thus, if a series of assays containing the same initial amount of biofilm 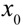 are performed using different volumes (*i.e.*, varying 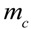), a plot of 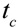 versus 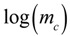 can be made and the specific growth rate can be extracted from the slope of the line for values of 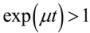: 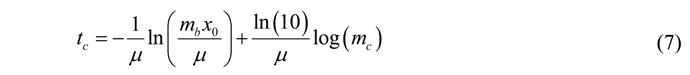 where the slope = ln(10)/*μ*. Once the specific growth rate is known, this information can be used to calibrate the results from antibacterial testing on the biofilm and thus quantitatively determine the log reduction. A schematic overview of the method alternative in which the volume of the assay media is varied is displayed in Figure 1. 

## 3. Experimental Section

### 3.1. Bacterial Culture Media and Biofilm Preparation

*Streptococcus mutans* (strain NCTC 10449) was employed in both planktonic and biofilm form in this study. *S. mutans* bacteria are known to initiate and dominate the growth of dental plaque [26]. Brain heart infusion (BHI) broth was used to culture planktonic *S. mutans*. The broth was prepared by dissolving 17.5 g BHI powder, 10 g peptone, 2 g glucose, 5 g NaCl and 2.5 g Na_2_HPO_4_ (all from Sigma-Aldrich, Steinheim, Germany) in 1 L distilled water followed by sterilization for 15 min at 121 °C. Biofilm culture media (BHIS broth) was made by adding 2 wt% sterile sucrose (VWR, Leuven, Belgium) to BHI broth. 

Sample disks for biofilm testing consisted of an adhesive resin made by mixing 2,2-bis[4-(2-hydroxy-3-methacryloxypropoxy)phenyl]-propane (BisGMA, Polysciences Europe GmbH, Eppelheim, Germany) and 2-hydroxyethyl methacrylate (HEMA, Sigma-Aldrich, Schnelldorf, Germany) in a 55/45 wt/wt ratio. Disks were cast in circular Teflon molds (diameter 8 mm, thickness 1 mm) and light-cured with 460 nm light for 20 s (BlueLEX GT1200, Monitex, Taiwan) under N_2_ flow.

To produce biofilm on the sample disks, *S. mutans* was first cultured to exponential phase in BHI broth (OD_600_ = 1.0) and then diluted in a container with 25 ml of BHIS broth to obtain a bacterial concentration of 10^6^ cfu/ml. Sample disks were cultured at the bottom of the container for 16 h at 37 °C. After cultivation the sample disks with biofilm were rinsed 5 times with 10 ml sterile Dulbecco’s phosphate buffered saline to remove the loosely attached bacteria [27]. One sample disk with 16-hour-old biofilm was fixed and sputter coated with gold/palladium (Polaron SC7640, Thermo VG Scientific, West Sussex, UK) and scanning electron microscopy (SEM) images were recorded with a LEO 1550 SEM (Zeiss, Oberkochen, Germany).

### 3.2. Specific Growth Rate of Planktonic S. mutans by OD Measurements

Planktonic *S. mutans* bacteria cultured to exponential phase in BHI broth were inoculated to 12 mL of BHIS broth and cultured statically at 37 °C. Every 30 min the culture was mixed well and a 300 μL aliquot was removed and turbidity measurements to assess the bacterial concentration were made using a multimode microplate reader (Infinite M200, Tecan) in absorbance mode at 600 nm. The specific growth rate of planktonic *S. mutans* in BHIS broth was calculated from these OD_600_ measurements as a function of time based on Equation (1). 

### 3.3. Metabolic Activity Assay (MAA)

The viability assay used in this study, based on the BioTimer assay [19] and hereafter referred to as the metabolic activity assay (MAA), evaluates the amount of viable bacteria with the help of a pH indicator. The accumulation of metabolic acid products causes a drop in pH of the assay, which is indicated by a change in color of the MAA from red to yellow due to the pH indicator phenol red. The MAA broth was prepared by adding 25 mg/L of phenol red (Sigma-Aldrich, Steinheim, Germany) to BHIS and adjusting the pH to 7.1.

### 3.4. Specific Growth Rate of Planktonic S. mutans by MAA Measurements

An MAA standard curve for planktonic *S. mutans* was determined by measuring the time for color change as a function of initial *S. mutans* concentration. Fourteen wells containing 2 mL of MAA broth were inoculated with *S. mutans* at bacterial concentrations ranging from 10 to 10^8^ cfu/mL. The sensitivity of the MAA, which is about 10 cfu/mL, is limited by deviations in diluting very small populations of cells. Incubation was performed at 37 °C without shaking, and color of the MAA broth was checked every 20 min. 

The initial *S. mutans* concentrations used for the MAA standard curve were set according to OD, which had been correlated to concentration by cfu counting. To ensure accurate correlation between OD and concentration, a 10 ml sample of the media containing bacteria was sonicated for 3 s at 20% intensity in order to separate the bacteria (Vibra-cell CV33 with tapered microtip 6300418, Sonics & Materials, Inc., Newtown, PA, USA) before culturing on an agar plate for cfu counting. Due to the possibility that the ultrasound treatment killed a small proportion of the cells, a comparison of the number of live cells before and after the ultrasonic treatment was made using a live/dead bacterial viability assay (L13152, Invitrogen, Eugene, OR, USA). It was found that 3% were killed due to the sonication, and thus a correction factor was applied to the OD-cfu correlation. 

Additionally, a series of ten MAA were performed on planktonic *S. mutans* (initial number of bacteria 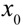 = 5 × 10^5^ cfu for all assays) in assay volumes ranging from 3 to 200 mL.

### 3.5. Standard Curves of Biofilm S. Mutans by MAA Measurements

A series of 6 MAA was performed on S. mutans in biofilm form in which the initial number of bacteria was controlled by varying the number of disks cultured with *S. mutans* biofilm from 1 to 32 disks in an assay volume of 10 mL. Finally, a series of 8 MAA was performed in assay volumes ranging from 1 to 50 ml in which each assay contained a single disk cultured with *S. mutans* biofilm.

## 4. Results and Discussion

### 4.1. Specific Growth Rate of Planktonic S. mutans by OD Measurements

**Figure 2:**
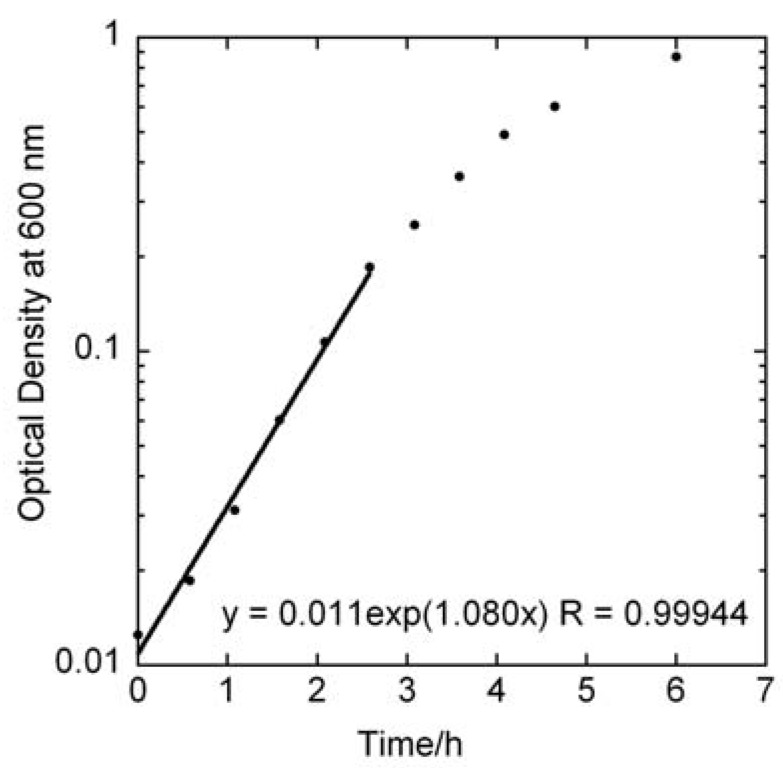
Specific growth rate of planktonic *S. mutans* as determined from OD_600 _measurements. A fit to Equation (1) for the initial growth phase is included (solid line), showing that the initial growth rate is *μ* = 1.08 h^−1^.

Figure 2 shows the OD_600_ of a planktonic *S. mutans* culture grown in BHIS broth as a function of time. Aliquots of culture were taken at 30 min intervals. The data in Figure 2 represents single measurements. When data points were recorded in triplicates the maximum variation in each point was found to be less than 5%. 

The decrease in growth rate after approximately 2.5 h indicates that the culture has entered another growth phase, possibly due to aggregation of bacterial cells. A fit to Equation (1) of the initial exponential growth phase portion of the OD curve (t < 2.5 h) gives a specific growth rate of 1.08 h^−1^. Since the OD is proportional to the concentration of bacteria, OD measurements are a standard method of determining the specific growth rate of bacteria. Note that the specific growth rate calculated here is relevant for *S. mutans* cultured in BHIS media, and is faster than the growth rate in BHI broth due to the added sucrose. OD measurements were made in BHIS media since this is the culture media used in the MAA.

### 4.2. Specific Growth Rate of Planktonic S. Mutans by MAA Measurements

**Figure 3:**
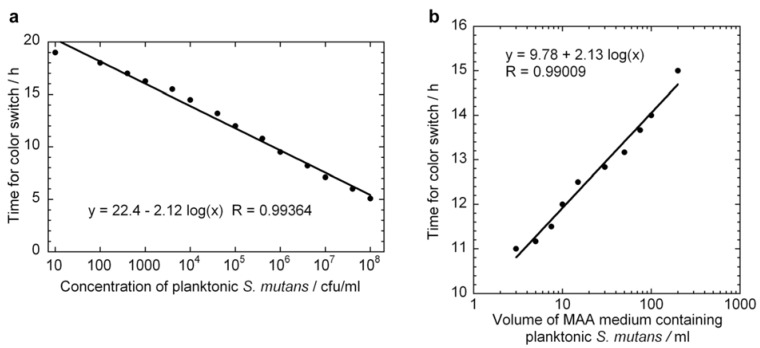
MAA measurements for planktonic *S. mutans* bacteria illustrating the time taken for the culture medium to switch color as a function of: (**a**) initial planktonic *S. mutans* concentration, and (**b**) assay volume for an initial planktonic *S. mutans* population of 5 × 10^5^ cfu. Curve fits to Equation (6) (panel a) and Equation (7) (panel b) are included as solid lines giving specific growth rates of 1.09 h^−1^ (a) and 1.08 h^−1^ (b).

Figure 3a displays single measurements of the time needed for the culture medium color to switch from red to yellow as a function of the initial concentration of planktonic *S. mutans* bacteria. The particular experiment was repeated three times in total and the variation in the slope of the fitted curve was found to be less than 2%. 

Time point 0 indicates the time at which incubation in the viability assay was started. From the figure, it is observed that the time it takes for the color of the culture medium to switch decreases logarithmically with increasing initial bacteria concentration in the entire concentration interval under study: When the initial *S. mutans* concentration is increased by a factor of 10, the color switching time decreases by 2.12 hours. From Equation (6) we find *μ* = 1.09 h^−1^, which is very close to the value that was calculated from the OD measurements. During the time interval prior to the color change of the MAA broth, the metabolic acid product (lactic acid) of the *S. mutans* accumulates [28]. The length of this time interval depends not only on the type and the initial concentration of bacteria in the medium, but also on systematic factors, like the buffering properties of the culture medium, the starting pH value and the type of nutrients the bacteria metabolize [28]. Therefore it is important to use the same batch of MAA broth to help ensure that the recorded time for color switch only depends on the initial concentration of *S. mutans* as shown in Figure 3a. 

The excellent agreement between the specific growth rate calculated from Figure 3a using Equation (6) and the specific growth rate calculated from OD measurements in Figure 2 suggests that the assumptions made in deriving Equation (6) are valid. The assumption that the bacteria exist in the exponential growth phase may not be valid at the start of the incubation in the MAA as the bacteria will likely transit an adjustment or lag phase, but if this delay is approximately constant for all tests, the slope of the 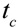 versus 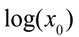 curve would remain unchanged. However, it is likely that these assumptions would not hold if too high of an initial concentrations of bacteria were used. From Figure 2 we can observe that the growth rate of the bacteria changes above OD values of about 0.2, which corresponds to a bacterial concentration of approximately 2 × 10^8^ cfu/ml. The highest initial concentration of bacteria used in Figure 3a was 10^8^ cfu/ml. Indeed, viability assay standard curves in several other studies [14,17,18,19] show such a log-linear relationship between the initial planktonic bacterial population and the time for a metabolic indicator to reach a certain level. In the MAA the dynamic range is very good as it shows an exponential relationship over seven orders of magnitude of initial bacterial concentration.

As indicated by Equation (7), if we vary the amount of metabolite needed to provide a certain indication (*i.e.*, by varying the volume of the assay), the specific growth rate can be determined from the slope of the 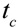 versus 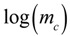 line. Figure 3b displays a series of ten MAA performed on planktonic *S. mutans* in assay volumes ranging from 3 to 200 ml in which the starting concentration of bacteria was identical in all tests. A logarithmic curve fit to the data and using Equation (7) gives *μ* = 1.08 h^−1^ in excellent agreement with both Figure 2 and Figure 3a.

### 4.3. Standard Curves and Specific Growth Rate of Biofilm S. Mutans by MAA Measurements

**Figure 4:**
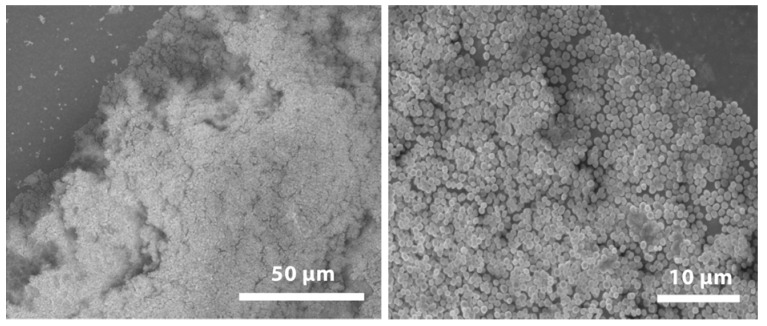
Scanning electron microscopy (SEM) images showing two magnifications of a 16-hour-old *S. mutans* biofilm attached on a sample disk.

Generally it is not possible to produce a standard curve for the MAA when testing biofilm viability because it is difficult to ascertain the initial number of bacteria in the biofilm. However, in this study we cultured biofilm onto small disks, and thus were able to approximately set the number of starting bacteria by varying the number of disks used in the MAA, assuming that the same number of viable bacteria was cultured on each disk. Figure 4 displays SEM images of a 16-hour-old *S. mutans* biofilm attached on the surface of a sample disk, in which multilayers of bacteria can be observed.

**Figure 5:**
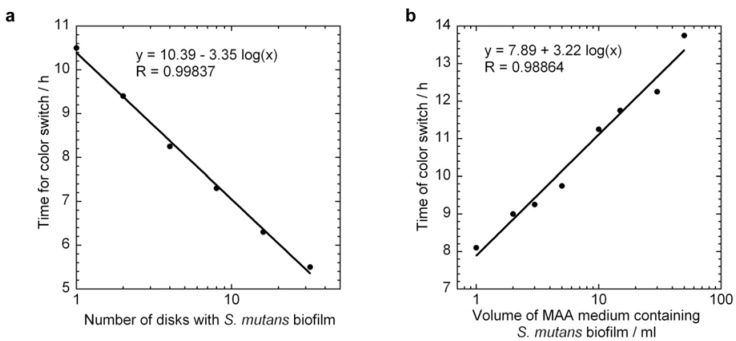
MAA measurements for biofilm *S. mutans* bacteria illustrating the time taken for the culture medium to switch color as a function of: (**a**) number of disks with *S. mutans* biofilm and (**b**) assay volume for an initial *S. mutans* biofilm on a single disk. Curve fits to Equation (6) (panel a) and Equation (7) (panel b) are included as solid lines giving specific growth rates of 0.687 h^−1^ (a) and 0.715 h^−1^ (b).

Figure 5a displays a series of 6 MAA measurements in which the number of disks inoculated in 10 ml of MAA broth was varied from 1 to 32 disks. This plot is similar to the standard curve in Figure 3a for planktonic bacteria, except that the number of biofilm bacteria in the different samples is not known. However, as detailed in the theory section above, the specific growth rate of the biofilm bacteria from this standard curve can be extracted using Equation (6) and used to quantitatively determine the log reduction in, for example, antibiotic susceptibility tests. By applying a logarithmic fit to the data points and applying Equation (6), a specific growth rate of 0.687 h^−1^ is found. This corresponds to a doubling time of 1 h compared to 38 min. for planktonic *S. mutans*. Note that the specific growth rate is derived without knowing the exact number of viable bacteria; instead it is the relative number of bacteria that is used to determine *μ*.

Figure 5b displays an alternate means of producing a standard curve for biofilm viability testing in which a series of 10 MAA of *S. mutans* biofilm on a single sample disk is tested in assay volumes ranging from 1 to 50 ml. A logarithmic curve fit to the data and using Equation (7) gives *μ* = 0.715 h^−1^ in very good agreement with Figure 5a. These two values determined for the specific growth rate of biofilm *S. mutans* bacteria differ less than 4% and give an average value of *μ* = 0.70 h^−1^.

This close agreement shows that we can determine the specific growth rate of biofilm bacteria by either varying the relative amount of biofilm on equivalent sample surfaces such as disks as in Figure 5a, or varying the assay volume while using the same starting amount of biofilm as in Figure 5b. As mentioned previously, although we can not determine the exact number of viable bacteria in a biofilm sample, the specific growth rate still allows us to calculate relative amounts, which is very useful in determining log reduction in antibiotic susceptibility testing or other bactericidal tests. 

It can be observed that the dynamic range of the MAA series in Figure 5 is much less than the dynamic range in Figure 3a. However, a much better estimation of the actual *μ* of the bacteria in biofilm form can be made with this method than if the standard curve from planktonic bacteria is used. This difference in *μ* can be quite important when quantifying the log reduction in bactericidal tests. For example, if the standard curve from planktonic bacteria, in which *μ* is higher than that for their biofilm counterparts, is used to calibrate the results from bactericidal tests on biofilms, an overestimation of the log reduction would result.

## 5. Conclusions

In this study we presented a scheme for quantitative determination of biofilm viability offering significant improvement over existing methods with metabolic assays. Two variations of a standard curve for biofilm bacteria were presented from which the specific growth rate of *Streptococcus mutans* bacterial biofilm was extracted. It was shown that this information could be used to more accurately quantify the relative number of viable bacteria in a biofilm in, for example, antibiotic susceptibility testing or other types of antimicrobial treatments. We found that the specific growth rate of *S. mutans* in biofilm form was 0.70 h^−1^, compared to 1.09 h^−1^ in planktonic form. Although this method was demonstrated using a *S. mutans* biofilm and a metabolic assay incorporating phenol red, it should be applicable to other bacteria types, as well as other metabolic assays, thus providing the opportunity to evaluate and compare new and existing antimicrobial treatments on biofilms.
